# Post-traumatic Growth Level and Its Influencing Factors Among Frontline Nurses During the COVID-19 Pandemic

**DOI:** 10.3389/fpsyt.2021.632360

**Published:** 2021-06-09

**Authors:** Xin Peng, Hui-zi Zhao, Yi Yang, Zhen-li Rao, De-ying Hu, Qin He

**Affiliations:** ^1^Cancer Center, Tongji Medical College, Union Hospital, Huazhong University of Science and Technology, Wuhan, China; ^2^Pediatric Department, Tongji Medical College, Union Hospital, Huazhong University of Science and Technology, Wuhan, China; ^3^Department of Nursing, Tongji Medical College, Union Hospital, Huazhong University of Science and Technology, Wuhan, China; ^4^Public Health Department, Tongji Medical College, Union Hospital, Huazhong University of Science and Technology, Wuhan, China

**Keywords:** COVID-19, frontline health worker, nurses, post-traumatic growth, influencing factors

## Abstract

**Objective:** To assess post-traumatic growth (PTG) level and explore its influence factors among frontline nurses during the COVID-19 pandemic.

**Methods:** From April 11th to 12th, 2020, a cross sectional study was conducted on 116 frontline nurses who had participated in fight against the COVID-19 in Wuhan city, China. General information and psychological discomfort were collected. Chinese version post-traumatic growth inventory with 20 items was applied to assess PTG level. Univariable analyses and multiple linear regression were performed to explore potential influencing factors of PTGI score.

**Results:** The average score of PTGI in frontline nurses was 65.65 ± 11.50. In univariable analyses, gender, age, education level, marital status, living with parents, professional title, working years and professional psychological support was not statistically associated with the PTGI score. In both univariable and multivariable analyses, having support from family members and friends, being psychological comfort and having children and increased the PTGI score significantly. The three factors only explained 3.8% variance.

**Conclusion:** Moderate PGT was observed in the frontline nurses who had battled against COVID-19. Social support and professional psychological intervention should be applied to further improve PTG level. Further studies with large sample size are required to explore more potential influencing factors.

## Background

In recent decades, the emergence of coronavirus has posed a huge threat on global health for causing significant mortality worldwide, such as severe acute respiratory syndrome (SARS-CoV) and Middle East Respiratory Syndrome Coronavirus (MERS-CoV) (1). In December 2019, the first case of coronavirus disease 2019 (COVID-19) emerged in Wuhan city, Hubei province, China (2).

The COVID-19 requires timely diagnosis and effective treatment to prevent progression to severe or critical infection and lower risk of death (3). Healthcare workers (HCWs) was the first-line fighters treating patients with COVID-19. Many HCWs in Wuhan city had been fighting against the COVID-19 pandemic for about 3 months. Increasing number of infected cases and uncertainty in the virus made HCWs under considerable workload and psychological pressure (4). A systematic review concluded high prevalence of post-traumatic stress symptoms (PTSS) related to the COVID-19 pandemic among HCWs and summarized potential predictors, such as young age, female and lack of social support (5).

Although a traumatic event can cause post-traumatic negative symptoms, the negative experience can be a “catalyst” for positive change, a growing number of studies showed positive post-traumatic growth (PTG) resulting from coping with trauma and an adaptive response to the adverse trauma (6, 7). PTG had been extensively studies in some natural disasters, such as earthquakes (8) and tsunami (9). HCWs may have great potential to develop PTG because of their professional characteristics. Nurses reported higher PTG score compared with social workers when working with war victims (10). During the COVID-19 pandemic, Kristine Olson and Martin Huecker emphasized the great significance to study PTG and its facilitators among HCWs (11, 12). A study conducted in February 2020 showed that 167 frontline nurses in Henan and Hubei, China, had demonstrated a moderate and above level of PTG during the early stages of the pandemic, meanwhile, the PTG level was associated with working years, self-confidence in frontline work, awareness of risk, psychological intervention, or training and deliberate rumination (13). Another large-scale survey conducted in April 2020 discussed relationship among burnout and PTG, influencing factors of PTG were not explored (14). However, little studies focused on the PTG level of nurses who had been locked in Wuhan city and had been working at frontline to against the pandemic from the beginning of the pandemic.

In this study, a selected tertiary Grade A hospital of Wuhan city was the first hospital to treat patients infected with COVID-19 from the beginning of the outbreak. More than 5,200 COVID-19 patients and 30,000 fever patients were admitted. This survey was conducted after the Wuhan city was unlocked at April 8th. It is of great significance to investigate post-traumatic growth level and its influencing factors among this population. Results from this study may help nursing managers identify nurses at risk of low PTG and develop systematic and effective intervention program.

## Methods

### Respondents

At January 23, 2020, the Wuhan city was blocked and the COVID-19 outbreak started. These nurses from a designated tertiary grade A hospital in Wuhan city were recruited to treat patients infected with COVID-19. At April 8th, 2020, the Wuhan city was unblocked. Until then, these nurses had been working in the isolation ward and had been living alone in a designated hotel to decrease transmission. We conducted this survey in the designated hospital from April 11th, 2020 to April 12, 2020. Inclusion criteria: (1) Had been participating in the frontline from beginning of the pandemic (2) working years ≥1 year, (3) agreed to participate in this survey.

This study was reviewed and approved by the Ethics Committee of the Union Hospital of Tongji Medical College, Huazhong University of Science and Technology [2020] Lunshenzi (0025); Special approval was obtained from the new coronavirus pneumonia emergency in 2020, project number 2020kfyXGYJ001.

### Measuring Instruments and Data Collection

A self-administered online questionnaire was developed and distributed by a QR code linked to questionnaire. Each question was required to be answered before submission, and the time consumed for each recorded was further inspected. The questionnaire consisted of three parts: (1) Informed consent and instruction, (2) basic characteristics, and (3) a Chinese version of Post-Traumatic Growth Inventory (PTGI).

The basic characteristics included age(years), gender (male/female), marital status (married/unmarried/divorced/widowed), education level (high school or below/college/undergraduate/postgraduate/doctor), professional title (general nurse/ nurse practitioner/supervisor nurse /chief nurse), working experience (years), whether you had children (yes/no), whether you lived with parents (yes/no), and whether you got support from family and friends during the epidemic (yes/no), and any physical discomfort during the epidemic (yes/no). If participants reported they had physical discomfort, they were required to check specific discomforts (yes/no for each item), including insomnia, gray hair/hair loss, weight loss, loss of appetite, irregular menstruation, Lumbar muscle strain/muscle soreness, coughing/sputum, and skin eczema.

Post-Traumatic Growth Inventory (PTGI) was developed by Tedeschi and Calhoun to assess PTG level (15). The original version included 21 items in 5 dimensions. In this study, a Chinese version with 20 items was adopted (16). Its Cronbach's α was 0.874. The item 18 “I am more firm in my religious belief” was deleted based on low correlation with total score and Chinese local culture. This scales consisted of 5 dimension, namely, Insights on life (6 items), personal strength (3 items), new possibilities (4 items), relationships with others (3 items), and self-transformation (4 items). The Likert scale was used, each score ranged from 0 to 5 for a total of 100 points. Higher score suggested higher level of PTG. A total score >60 or average item score >3 indicated moderate and higher levels of PTG (17, 18).

### Statistical Analysis

Age was classified into three categories, 20~30, 31~40, and 41~50 years. Work experience was divided into three types, <3, 3~8, and >8 years. With the limitation of small sample size, one category with few number in basic variable was combined based on medical knowledge. For PTGI score, descriptions were conducted for total score, 5 domains, and 20 items.

Categorical variables were described as frequency and percentage. Continuous variables were expressed as mean ± standard deviation or median (interquartile range) based on normality test. We performed group comparisons on total PTGI score for all basic characteristics. Both normality and homogeneity of variance were tested, Student' *t*-test or Wilcoxon rank-sum test was applied for two groups, analysis of variance or Kruskal-Wallis *H*-test were conducted for more than two groups. In multivariable regression, all basic characteristics were included, stepwise linear regression analysis was used to select potential effects of basic characteristics on PTGI. In the regression, binary variable (yes/no) of any physical discomfort was included instead of each specific discomfort.

All statistical analyses were conducted using SPSS version 19.0 (SPSS Inc., Chicago, IL). *P* < 0.05 (2-sided) was considered statistically significant.

## Result

A total of 116 participants completed the questionnaires. After checking the filling time and missing values, no record was excluded, finally, 116 participants were included for final analysis. The average age was 34.07 years, 40% were younger than 30 years and the majority of participants was female (106, 91.40%). The average of total PTG score was 65.65 ± 11.50 and the average score of 20 items were 3.28 ± 0.57. Insights on life had the highest average score (median, 3.67), followed by personal strength, relationship with others, self-transformation and new possibilities, see Table 1. For each item, the top 3 items were item 13 (I can cherish each day better), item 15 (I have more sympathy for others) and item 2 (I have a better understanding of my life value). The last 3 items were item 14 (This event brought me a new opportunity), item 16 (I spent more energy on inter personal relationships), and item 3 (I developed a new interest).

**Table 1:** Total score of Post-traumatic growth inventory and its 5 dimension and their average score of items.

**Post-traumatic growth**	**Score Mean ± SD/Median (Q1, Q3)**	**Average score of items Mean ± SD/Median (Q1, Q3)**
Post-traumatic growth total score, 20 items	65.65 ± 11.50	3.28 ± 0.57
**5 domains**		
Insights on life, 6 items	22.00 (20.00, 25.00)	3.67 (3.33, 4.17)
Personal strength, 3 items	10.00 (9.00, 11.00)	3.33 (3.00, 3.67)
New possibilities, 4 items	12.00 (10.00, 13.00)	3.00 (2.50, 3.25)
Relationship with others, 3 items	9.00 (8.00, 10.00)	3.00 (2.67, 3.33)
Self-transformation, 4 items	12.00 (11.00, 14.00)	3.00 (2.75, 3.50)

*SD, standard deviation; Q1, the first quartile; Q3, the third quartile*.

In group comparison for basic characteristics, participants having child/children reported significantly higher PTG score than those without child/children, 67.27 ± 12.13 vs. 61.89 ± 8.94, *P* < 0.001. Compared with participants without any physical discomfort during the epidemic, participants who reported physical discomfort had higher PTG score (66.72 ± 11.52 vs. 61.05 ± 10.45, *P* = 0.036). Meanwhile, significantly higher PTG was observed in participants who got support from family and friends during the epidemic, 65(58, 74) vs. 59 (55, 63), *P* = 0.043. However, no significant difference existed in PTG score as related to gender, age group, marital status, education level, professional title, working experience group, and living with parents before the epidemic (Table 2).

**Table 2:** Univariable analysis of basic characteristics on post-traumatic growth.

**Variable**	**Number**	**PTG Score**	**F/t/z**	***P***
Gender			0.27	0.785
Female	106	65.56 ± 11.60		
Male	10	66.60 ± 10.84		
Age (years)			0.64	0.532
20–30	46	64.17 ± 10.35		
31–40	50	66.76 ± 12.40		
41–50	20	66.25 ± 11.88		
Marital status			1.75	0.083
Married	91	66.62 ± 11.89		
Unmarried and others	25	62.12 ± 9.33		
Education			0.40	0.693
College and below	14	66.50 ± 7.92		
Undergraduate and above	102	65.53 ± 11.93		
Professional title			0.27	0.765
Nurse	18	63.83 ± 11.77		
Nurse practitioner	63	65.87 ± 11.24		
Supervisor nurse and higher	35	66.17 ± 12.50		
Working experience (years)			0.76	0.470
< 3	42	64.64 ±10.78		
3–8	49	67.18 ± 12.34		
≥9	25	64.32 ± 11.05		
Whether you have children			2.66	0.009
Yes	81	67.27 ± 12.13		
No	35	61.89 ± 8.94		
Whether you live with parents before the epidemic			0.66	0.512
Yes	52	64.87 ± 11.78		
No	64	66.28 ± 11.32		
Any physical discomfort during the epidemic			2.11	0.036
Yes	94	66.72 ± 11.52		
No	22	61.05 ± 10.45		
Getting support from family and friends during the epidemic^a^			2.02	0.043
Yes	100	65(58, 74)^b^		
No	16	59 (55, 63)^b^		

a *Wilcoxon rank-sum test*;

b *median (the first quartile, the third quartile)*.

*PTG, Post-traumatic growth*.

In stepwise linear regression, all basic characteristics were included, but only having children, any physical discomfort and getting support from family and friends during the epidemic were kept in morel and independently and significantly increased the PTG score, 5.34 (95%CI, 0.87–9.90), 5.68 (95%CI, 0.36–10.99), and 9.82 (95%CI, 0.41, 19.24), respectively (Table 3). However, the three included characteristics only explained the 3.8% variation of PTG score (adjusted *R*^2^ = 0.038), which indicated that other key factors were not included.

**Table 3:** Multiple stepwise regression of post-traumatic growth and related factors.

**Variable**	**Coefficient (95% CI)**	***t***	***P***
**Whether you have children**			
Yes	5.34 (0.87, 9.90)	2.36	0.02
No	Reference	–	–
**Any physical discomfort during the epidemic**			
Yes	5.68 (0.36, 10.99)	2.12	0.04
No	Reference	–	–
**Getting support from family and friends during the epidemic**			
Yes	9.82 (0.41, 19.24)	2.07	0.04
No	Reference	–	–

At last, the description of specific physic discomforts among 94 participants was reported in Table 4. The main symptom is insomnia (59.5%). About one in five nurses experienced gray hair/hair loss, weight loss, and loss of appetite. Meanwhile, about 10% nurses suffered from irregular menstruation.

**Table 4:** The prevalence of physical discomfort among 116 participants.

**Symptoms of discomfort**	**Frequency**	**Percentage**
Insomnia	69	59.48%
Gray hair/hair loss	31	26.7%
Weight loss	28	24.1%
Loss of appetite	25	21.6%
Irregular menstruation	11	10.4%
Lumbar muscle strain/muscle soreness	2	1.72%
Coughing/Sputum	2	1.72%
Skin eczema	1	0.9%

## Discussion

Sudden emergency of the COVID-19 epidemic can be understood as a traumatic event which may trigger a PTSD-like responses and mental problems. In our study, these frontline nurses had been working in the epidemic center since the COVID-19 outbreak in Wuhan city. After Wuhan city was unlocked, the average score of PTG, as positive effect of the COVID-19 epidemic, was 65.65. It was similar to 70.53 in Pan's study (13). It suggested frontline nurses experienced a moderate and high growth after the epidemic.

Although the Wuhan city has many medical resources, including four tertiary A hospitals, a large number of patients have flowed into the hospital after the outbreak and it resulted in an apparent deficiency of medical resources. Many local nurses had been fighting against the COVID-19 for about 3 months since then. High-intensity and high-risk work required them to maintain resilience. During wok, these frontline nurses had to face many critically ill patients and deaths. After work, they were isolated in a single room, unable to meet with family and friends, and maintained a social distance with others. However, higher scores in “Treasure every day,” “I have more sympathy for others,” and “Better understanding of my life value” suggested that the experience make them realized the value of life. These nurses experienced the epidemic from the block to unblock, which could greatly affirm their efforts.

In this study, having children increased PTG level. “Mother being strong” is a public opinion on mothers. The duties and role of mothers make them more brave and strong when facing difficulties and challenges. A psychological research on frontline nurses showed that the identity of “mother” shows a higher level of post-traumatic growth after trauma (19). Appearance of physical discomfort during the epidemic also elevated the PTG level. The main symptoms of COVID-19 were similar to other common diseases, any physical discomfort during the fighting might be considered as additional negative event, which caused the nurses to suspect being infected. They might feel lucky if these discomforts relieved, which promoted the positive changes eventually. Moreover, an improved physical condition could help nurses cope with stress and reduce the psychological burden. Higher PTG was observed in nurses who got support from family and friends. Social support can turn trauma into growth by activating the cognitive process that promotes PTG (20). A study on victims of the Sewol Ferry disaster showed that social support was positively associated with PTG level (21). During the epidemic, two studies on Chinese healthcare workers found that social support relieved psychological pressure and promote mental health (22, 23). However, it should be noted that the three factors only explained a small part of variation of PTG score. The Wuhan city where the participants located had just been unlocked, participants were still working at frontline and had not enough time to reflect deeply, and the potential characteristics affected the PTG level slightly.

Overall, the frontline nurses reported moderate PTG level. Post-traumatic depreciation, inverse of PTG, can coexist with PTG in the aftermath of Trauma (24). Nursing administrators should make effective strategies to further improve PTG among frontline nurses. Promotors for PTG had been summarized by another systematic review by Charlotte Henson, such as sharing negative emotions and positive reappraisal (25). A novel intervention program had been developed to improve nurses' PTG significantly (26). Based on results in this study, three strategies can be recommended. Firstly, increasing social support. The importance role of social support from family and friends during MERS-CoV epidemic and COVID-19 epidemic has been emphasized (27, 28). We should encourage family members, friends, and colleagues to maintain communication and communication with frontline nurses as much as possible. Item 14 “This event brought me a new opportunity” got the lowest score, it was significant to increase the rewards and give preferential policy for title evaluation and recruitment for these frontline nurses. Meanwhile, the media should cooperate with the hospital to guide the public correctly, such as reducing the panic caused by the fear of being infected by healthcare work. Secondly, regular screening for nurse with low PTG level and organizing professional psychological intervention. It can help frontline nurses eliminate fear, reduce psychological burden, and relieve work pressure. Setting up an anti-epidemic narrative nursing team can be an appealing method to conduct online psychological assistance and offline psychological assistance. Thirdly, no one is sure when the next outbreak will be. When facing stress, the different coping styles adopted by healthcare workers may have an important effect on mental health (29). Nurses with positive coping, appropriate social experience and psychological maturity should be recruited to the frontline. In addition, mindfulness decompression therapy is an effective strategy for relieving high-intensity stress and strengthening ability to regulate emotions (30).

The survey was a cross-sectional study with small sample size. New psychological problems may be revealed over time, meanwhile, this study only investigated a tertiary Grade A general hospital in Wuhan, representativeness of the sample was limited. Only three influencing factors were found with low explanation, future research should continue to elucidate potential factors that are predictive of PTG level. Meanwhile, a large longitudinal study in different regions was suggested to further explore PTG level and its change profile, and more potential influencing factors, which can formulate effective measures to promote nurses' PTG.

## Conclusion

In this study, we observed moderated PGT level among these frontline nurses who had battled against COVID-19 in Wuhan city for more than 3 months. Having children, physical discomfort and getting support from family and friends during the epidemic were three influencing factors. Social support and professional psychological intervention should be applied to further improve PTG level. Moreover, further multicenter longitudinal studies with large sample size are required.

## Data Availability Statement

The original contributions presented in the study are included in the article/supplementary material, further inquiries can be directed to the corresponding author/s.

## Ethics Statement

The studies involving human participants were reviewed and approved by Medical Ethics Committee. The patients/participants provided their written informed consent to participate in this study.

## Author Contributions

XP initiated and conceived this research article with her nursing team, collected data and supported with the first-line nurses' interviews, supervised, gave suggestions, and has involved in the original article writing. HZ has involved in contributing article with English version and translated the article. YY has involved in original article writing with Chinese version and supported the interview missions. ZR has involved in original article writing with Chinese version and supported the interview missions. DH has supervised our interviews and gave some professional advices to our article. QH has gave some professional advices to our article. All authors contributed to the article and approved the submitted version.

## Conflict of Interest

The authors declare that the research was conducted in the absence of any commercial or financial relationships that could be construed as a potential conflict of interest.
